# Enhanced Differentiation Capacity and Transplantation Efficacy of Insulin-Producing Cell Clusters from Human iPSCs Using Permeable Nanofibrous Microwell-Arrayed Membrane for Diabetes Treatment

**DOI:** 10.3390/pharmaceutics14020400

**Published:** 2022-02-12

**Authors:** In Kyong Shim, Seong Jin Lee, Yu Na Lee, Dohui Kim, Hanse Goh, Jaeseung Youn, Jinah Jang, Dong Sung Kim, Song Cheol Kim

**Affiliations:** 1 Asan Institute for Life Sciences, Asan Medical Center, University of Ulsan College of Medicine, Seoul 05505, Korea; shimiink@gmail.com (I.K.S.); ynlee426@naver.com (Y.N.L.); standard0130@naver.com (H.G.); 2 Department of Medical Science, Asan Medical Institute of Convergence Science and Technology (AMIST), Asan Medical Center, University of Ulsan College of Medicine, Seoul 05505, Korea; 3 Department of Mechanical Engineering, Pohang University of Science and Technology (POSTECH), 77 Cheongam-ro, Nam-gu, Pohang 37673, Korea; leeseongjin@postech.ac.kr (S.J.L.); dheekim328@postech.ac.kr (D.K.); jasonyoon@postech.ac.kr (J.Y.); jinahjang@postech.ac.kr (J.J.); 4 School of Interdisciplinary Bioscience and Bioengineering, Pohang University of Science and Technology (POSTECH), Cheongam-ro, Nam-gu, Pohang 37673, Korea; 5 Department of Convergence IT Engineering, Pohang University of Science and Technology (POSTECH), 77 Cheongam-ro, Nam-gu, Pohang 37673, Korea; 6 Institute for Convergence Research and Education in Advanced Technology, Yonsei University, 50 Yonsei-ro, Seodaemun-gu, Seoul 03722, Korea; 7 Department of Chemical Engineering, Pohang University of Science and Technology (POSTECH), 77 Cheongam-ro, Nam-gu, Pohang 37673, Korea; 8 Department of Surgery, Asan Medical Center, University of Ulsan College of Medicine, 88 Olympic-ro 43-gil, Songpa-gu, Seoul 05505, Korea

**Keywords:** iPSC, insulin-producing cells, diabetes, cell cluster, electrospinning, microwell, nanofibrous membrane

## Abstract

Although pancreatic islet transplantation is a potentially curative treatment for insulin-dependent diabetes, a shortage of donor sources, low differentiation capacity, and transplantation efficacy are major hurdles to overcome before becoming a standard therapy. Stem cell-derived insulin-producing cells (IPCs) are a potential approach to overcoming these limitations. To improve the differentiation capacity of the IPCs, cell cluster formation is crucial to mimic the 3D structure of the islet. This study developed a biodegradable polycaprolactone (PCL) electrospun nanofibrous (NF) microwell-arrayed membrane permeable to soluble factors. Based on the numerical analysis and experimental diffusion test, the NF microwell could provide sufficient nutrients, unlike an impermeable PDMS (polydimethylsiloxane) microwell. The IPC clusters in the NF microwells showed higher gene expression of *insulin* and *PDX1* and insulin secretion than the PDMS microwells. The IPC clusters in the NF microwell-arrayed membrane could be directly transplanted. Transplanted IPC clusters in the microwells survived well and expressed PDX1 and insulin. Additionally, human c-peptide was identified in the blood plasma at two months after transplantation of the membranes. The NF microwell-arrayed membrane can be a new platform promoting IPC differentiation capacity and realizing an in situ transplantation technique for diabetic patients.

## 1. Introduction

Diabetes is a cause of various systemic complications, such as heart disease, kidney failure, stroke, and diabetic neuropathy, along with a shortened life expectancy, leading to substantial medical costs worldwide [1]. In insulin-dependent diabetes, exogenous insulin injections are the standard treatment to reduce hyperglycemia. However, general insulin injections can cause severe hypoglycemia and cannot prevent long-term complications because they do not allow for precise blood glucose control such as that provided by a healthy pancreas [2,3,4]. Precise real-time diabetes control tailored to the blood glucose level is important to prevent complications of diabetes [5,6]. Thus, transplantation of the pancreas and pancreatic islets is a potentially curative treatment for diabetes.

Unlike pancreas transplantation, pancreatic islet transplantation is relatively simple and non-invasive. In 2000, the Edmonton group reported seven patients who successfully became insulin-independent one year after islet transplantation [7]. However, only 20% of these patients remained insulin-independent by five years, and the remaining 80% needed insulin injections again [8]. Although pancreatic islet transplantation is an ideal treatment, there are still many hurdles it must overcome before becoming a standard therapy. Its significant issues include a shortage of donor sources and a low engraftment efficacy of the islets after transplantation [9].

Previously, transplanted islets could only be obtained from cadaveric donors [10]. With the recent technological advances in stem cells and molecular biology, insulin-producing cells differentiated from stem cells are being developed as an alternative source of islets [11,12]. However, they still exhibit limited glucose control in vivo due to their low physiological functions, different from natural islets. It is well known that the cell–cell aggregation structure of islets is essential for maintaining their physiological functionality. Therefore, various studies on differentiated islets have attempted to mimic the morphological characteristics of natural islets to enhance their insulin-producing functionality [13,14,15]. Among the numerous approaches, aggregating the differentiated islets into 3D structures has shown significant effects on enhancing their insulin-producing functionality, and several approaches have been developed to generate aggregates of differentiated islets. Recently, the microwell array was highlighted as providing an easy and rapid way to create aggregates of the desired size. However, conventional microwells exhibit limited nutrients and oxygen supply because they are made of impermeable material except on the upper surface. The limited nutrients and oxygen supply in a microwell array can potentially disrupt the differentiation process of stem cells into pancreatic islets or undermine the insulin-producing functionality of the differentiated islets.

Currently, in clinical practice, islets are transplanted into a blood vessel (portal vein) under the liver of diabetic patients [16]. Islet infusion through the hepatic portal vein causes an immediate blood-mediated inflammatory response and apoptosis of the islet cells. Additionally, portal hypertension, hemorrhage, and thrombosis can occur during portal vein injection, which may cause severe complications [17,18,19]. To solve these problems, several alternative transplantation sites such as subcutaneous sites or the liver surface, peritoneum, and omentum have been proposed [20,21,22,23]. The differentiated islets first need to be harvested from the microwell array because the microwell array is not transplantable. However, the transplanted islets without any scaffolds are quickly swept away or degraded rapidly in the tissue of patients, resulting in low transplantation efficacy. In this regard, transplantable scaffolds are frequently utilized to enhance the transplantation efficacy, helping to maintain the 3D structure of the islets after the transplant. Recently, functional and transplantable scaffolds have been developed based on tissue engineering technologies such as cell sheet engineering, 3D bioprinting, functional hydrogels, or polymer manufacturing.

A sheet or membrane can be implanted directly and attached to the surface of various organs. Electrospinning is a method that can easily manufacture nanofibrous membranes by spinning various biomaterials and polymers using electric charges [24]. Various electrospun medical devices, drug delivery systems, and implants have been developed. This study presents a permeable nanofibrous (NF) microwell-arrayed membrane, which addressed the aforementioned limited differentiation capacity and realized in situ transplantation of differentiated insulin-producing cell clusters without harvesting. Gas and soluble factors were permeated because the NF microwell-arrayed membrane was fabricated using a matched-mold process on an electrospun permeable NF membrane. Numerical analysis and a diffusional transport test simulated and demonstrated that the fabricated NF microwell provides more nutrients to iPSC clusters than a typical impermeable PDMS microwell. The IPC differentiation capacity under different culture conditions was compared by analyzing the pancreas-related gene expression and insulin secretion. In addition, IPC clusters differentiated from iPSCs in a permeable NF microwell-arrayed membrane were transplanted subcutaneously and to the surface of organs such as the liver and peritoneum to evaluate implantability and function in vivo.

## 2. Materials and Methods

### 2.1. Fabrication of the NF Microwell-Arrayed Membrane

#### 2.1.1. Electrospinning for Fabrication of the NF Membrane

Polycaprolactone (PCL; Mn = 80,000 g mol^−1^), chloroform, and methanol were purchased from Sigma–Aldrich (St. Louis, MO, USA) and used as received. The electrospinning solution was prepared by dissolving PCL in a mixture of chloroform/methanol (3:1 vol:vol) to obtain a concentration of 7.5 wt %. The prepared PCL solution was then loaded into a 5 mL gas-tight syringe (Hamilton, Reno, NV, USA) and fed through a 23-gauge metal needle placed 10 cm above a ring collector with a diameter of 20 mm. Subsequently, electrospinning was performed using a commercial electrospinning machine (ESR200R2, NanoNC, Seoul, Korea). The flow rate was set to 1 mL h^−1^, and a high voltage of 15 kV was applied between the metal capillary and ring collector for electrospinning. A relative humidity of 50–60% and temperature of 20–25 °C were maintained during the electrospinning. As-spun PCL nanofibers were deposited on the grounded ring collector with a random orientation, resulting in the formation of an NF membrane. The prepared flat NF membrane was transferred to a poly(methyl methacrylate) (PMMA) ring covered with an adhesive in a free-standing configuration.

#### 2.1.2. Fabrication of a Pair of Male and Female Matched Molds

The NF microwell-arrayed membrane was fabricated by a matched-mold forming process with the electrospun flat NF membrane. The female mold for the desired shape of the microwell array was prepared using a micromachining machine (EGX-360, Roland, Irvine, CA, USA) with a tapered ball-end milling cutter on a PMMA substrate (AcrylChoika, Seoul, Korea). The polydimethylsiloxane (PDMS) male mold was prepared by PDMS replica molding against the female mold. Briefly, an uncured mixture of PDMS and curing agent in a weight ratio of 5:1 (Sylgard 184, Dow Corning, Midland, MI, USA) was poured into the female mold and baked in a convection oven at 55 °C for 12 h.

#### 2.1.3. Fabrication of the NF Microwell-Arrayed Membrane Using a Pair of Male and Female Matched Molds

The flat PCL NF membrane transferred to the PMMA ring as described in Section 2.1.1 was placed between the male and female molds. The movement of the male mold was controlled by a motorized stage (KS162-200, Suruga Seiki, Shizuoka, Japan), which moved at a constant speed of 2.0 mm s^−1^ and was integrated with a single point load cell (BCL-2L, CAS, Yangju, Korea) to validate the compressive force. The displacement of the male mold to match the female mold applied compressive force to the flat NF membrane. After maintaining the matched position of the male mold for 10 s and moving it to the original position, the deformed NF membrane was carefully detached from the female mold, which resulted in the NF microwell-arrayed membrane, including 165 microwells. The NF membrane was finally integrated with the bottom opening of a custom-designed 12-well insert wall with no membrane, produced by an injection molding machine (SE50D, Sumitomo, Tokyo, Japan). In detail, a ring-shaped double-sided tape (inner diameter of 12 mm and outer diameter of 15 mm; 467 MP, 3 M, Saint Paul, MN, USA) was fabricated using a laser cutter (ML-7050A, MachineShop, Paju, Korea) and attached to the bottom opening of the insert wall. The PMMA ring with the NF microwell was then integrated with the insert wall with the help of double-sided tape. The microwell insert was designed to be immersed in the culture medium in a conventional 12-well plate. Before the cell culture, the remaining organic solvent was removed by a freeze-dryer for 48 h and sterilized by low-temperature EO gas for 36 h.

### 2.2. Characterization of the NF Microwell

#### 2.2.1. Shape, Nanofibrous Structure, and In-Plane Porosity of the NF Microwell

The top view of the NF microwell-arrayed membrane integrated into the custom-designed 12-well insert well was examined by obtaining photographs using a DSLR camera (EOS650, Canon, Tokyo, Japan). A more detailed overall view was also taken using SEM images acquired by a field-emission scanning electron microscope (FE-SEM, SU6600, Hitachi, Tokyo, Japan). The structure of the interconnected nanofibers was examined using a high magnification of the SEM image. Furthermore, to characterize the in-plane porosity of the interconnected nanofibers, the magnified SEM images were converted to binary images through the threshold process in ImageJ software (NIH, Bethesda, MD, USA), and the in-plane porosity of the NF microwell was measured by calculating the area fraction of the pores and nanofibers using ImageJ software. Subsequently, a cross-sectional image of a microwell was obtained by optical microscopy (Eclipse 80i, Nikon, Tokyo, Japan) and confocal microscopy (FV3000, Olympus, Tokyo, Japan) after staining the NF microwell array with rhodamine 6G (5 mg mL^−1^ in PBS) for 6 h at room temperature.

#### 2.2.2. Diffusional Transport Test through the NF Microwell-Arrayed Membrane

The diffusional transport of the soluble factors through the NF wall was experimentally demonstrated by utilizing red dyes (Edentown, Incheon, Korea) composed of maltodextrin, for which the molecular weight was from 9 to 155 kDa. After placing 2 mL of the 200 µg mL^−1^ red dye on the basolateral side of the NF microwell and 2 mL of the water apical side, the diffusional transport time was evaluated by obtaining photographs using the DSLR camera. To confirm the soluble factors of the iPSC clusters in the microwells, we also confirmed the GFP expression of the iPSCs on the PDMS microwells or NF microwells using fluorescence microscopy after the transduction of adenoviral GFP expression vectors (Ad-GFP, Vector Biolabs, Malvern, PA, USA) with MOI 200 for 48 h.

### 2.3. Numerical Analysis of the Glucose Concentration Surrounding the iPSC Cluster in the NF and Impermeable Microwell

The spatiotemporal glucose concentration around the iPSC cluster was numerically simulated using COMSOL Multiphysics software (version 5.0, Burlington, MA, USA). All geometries and dimensions used in the numerical simulation were reflected by those used in the experimental setup. At the bottom of both the NF and impermeable microwell, a spherical void space equivalent to the average diameter of the iPSC cluster (300 µm) was introduced to simulate the iPSC cluster. The initial glucose concentration was set to 11.1 mol m^−3^, equivalent to the corresponding concentration in the utilized RPMI1640 cell culture medium (Gibco BRL, Grand Island, NY, USA). Along the boundary of the spherical void, the glucose consumption rate was calculated to be 0.267 mol m^−3^ s^−1^ based on the experimentally measured glucose consumption rate of the pancreatic islet spheroids reported previously [25]. As the diffusion coefficient of the glucose concentration in the culture medium was 580 µm^2^ s^−1^, it was simulated in the present simulation. The porosity of the NF microwell was estimated to be 0.046 based on the measured in-plane porosity (Figure S1), as described in Section 2.2.1, to predict the solute diffusivity in a porous material using the Millington−Quirk model. On the contrary, the porosity of the impermeable microwell was set to 0.

### 2.4. Cell Culture and Differentiation

Human iPSC lines (WTC-11: Coriell Institute, Camden, NJ, USA) were maintained in Stem-MACSiPS-Brew XF, Human medium (MiltenyiBiotec, Auburn, CA, USA) containing 10 μM Y-27632 (Selleck Chemicals, Pittsburgh, PA, USA) on Vitronectin (Thermo Fisher Scientific, Waltham, MA, USA) coated dishes. The cell culture was carried out at 37 °C under 5% CO_2_ in the air. Human iPSCs were differentiated into insulin-producing cells using the modified three-stage protocol described by Lee et al. [26]. Stage one is as follows: the iPSCs were induced to definitive endoderm in RPMI 1640 medium (Gibco, USA) containing 2% FBS (Gibco BRL, Grand Island, NY, USA), 100 ng/mL activin A (PeproTech, Cranbury, NJ, USA), 3 μM CHIR99021 (Sigma Aldrich, St. Louis, MO, USA), and 10 μM Y-27632 for 24 h and then in fresh RPMI 1640 medium (Gibco, USA) containing 2% FBS (Gibco, USA), 100 ng/mL activin A, and 10 μM Y-27632 for 2d. Stage two is as follows: the cells were induced into pancreatic progenitor cells with Improved MEM Zinc Option medium (Gibco, USA) containing 1% B27 minus insulin (Gibco BRL, Grand Island, NY, USA), 1 μM dorsomorphin (Torcis Bioscience, Bristol, Avon, UK), 2 μM retinoic acid (Sigma Aldrich, USA), 10 μM SB431547(Selleck Chemicals, Pittsburgh, PA, USA), 0.25 μM SANT-1 (Sigma Aldrich, USA) for 7 days. On day 4, the cells were harvested, and 10^6^ cells were replated in a 6-well culture plate, PDMS microwell, or NF microwell. We used a commercially available microwell (StemFIT 3D, Microfit Co., Hanam-si, Gyeonggi-do, Korea). We coated the plates with 3% BSA for 2 h before seeding to prevent cell attachment to the surface of the microwell, which would induce cell aggregation in the microwells. Stage three is as follows: the cells were induced into insulin-producing cells (IPC), which were cultured for 11 days in Improved MEM Zinc Option medium containing 1% B27 minus insulin, 10 μM forskolin (Sigma Aldrich, St. Louis, MO, USA), 10 μM dexamethasone (Selleck Chemicals, Pittsburgh, PA, USA), 10 mM nicotinamide (Sigma Aldrich, USA), 10 μMExendin-4 (Torcis Bioscience, Bristol, Avon, UK), and 1 μM triiodothyronine (T3, Sigma Aldrich, St. Louis, MO, USA). The medium was replaced every two days.

### 2.5. Monitoring of Cluster Formation of Cells in the PDSM and NF Microwells

On day 4, the cells were harvested and seeded again in microwells to make a cell cluster as described in Section 2.4. Because the PDMS microwell is transparent, we checked the cluster formation and migration using optical microscopy. However, it is difficult to observe the NF microwell using an optical microscope, so the samples were fixed with a 2.5% glutaraldehyde solution at determined time points, and the cluster shape was confirmed through an SEM image. For the SEM analysis, the cell-seeded NF microwells were pre-treated inOsO4 solution and dehydrated using a series of chilled ethanol solutions (70, 80, 90, 95, and 100%) and then placed into a 1,1,1,3,3,3-Hexamethyl silazane solution for 1 h. The samples were dried at room temperature. The morphology of the cell-seeded NF microwells was observed using SEM (AIS2000C, Seron Technologies, Uiwang-si, Gyeonggi-do, Korea).

### 2.6. Quantitative Real-Time PCR (qPCR)

The total RNA was extracted from the IPCs on days 6, 10, and 15 using TRIzol (Thermo Fisher Scientific, Waltham, MA, USA) according to the manufacturer’s instructions. The IPC clusters in the microwells were also broken mechanically using a syringe. The cDNA was synthesized from a 1μg RNA template by an oligo-dT primer using a SuperScript III First-Strand Synthesis System (Thermo Fisher Scientific, Waltham, MA, USA) at 50 °C for 60 min and 70 °C for 15 min. Real-time PCR was performed using the LightCycler 480 SYBR Green I Master Mix (Roche Applied Science, Mannheim, Germany) in a LightCycler^®^ 480 II real-time thermal cycler (Roche Applied Science, Mannheim, Germany). The primer sets for the pancreas-related genes are listed in Table 1. The samples were amplified according to the following procedure: polymerase activation at 95 °C for 5 min, followed by 40 cycles of annealing/extension/detection at 95 °C for 10 s, 57 °C for 45 s, and 72 °C for 60 s. All gene expression was normalized to the *GAPDH* housekeeping gene, and relative quantification was performed using the delta CT method. Statistical analyses were conducted using the t-test and reported in all figures.

### 2.7. Insulin Production

On days 17, 19, and 21, we confirmed insulin secretion by the IPCs in the cell culture medium. The insulin content of the medium was measured using a commercial ultrasensitive insulin ELISA Kit (Alpco, Salem, NH, USA) according to the manufacturer’s instructions. The absorbance was measured at 450 nm, using a Microplate Absorbance Reader (Sunrise, Tecan Austria GmbH, Salzburg, Austria). To confirm the immunohistochemistry image of the insulin and PDX1 expression in the IPC clusters, IPCs in the NF microwell were fixed in 4% paraformaldehyde (PFA; Merck, Darmstadt, Germany) for 10 min at 4 °C and washed twice with phosphate-buffered saline (PBS) on day 21. The membranes were embedded in Tissue-Tek (Sakura Finetek, Torrance, CA, USA) and sectioned (6 μM) to acquire frozen tissue blocks. The cells were permeabilized with 0.1% Triton X-100 at 25 °C for 10 min and washed three times with PBS. For antibody blocking, the cells were incubated in 3% bovine serum albumin for 1 h at room temperature. The primary antibody was incubated with anti-guinea pig insulin (1:200; Abcam, Cambridge, UK) and rabbit anti-PDX1 (1:200; Abcam, MA, USA). The primary antibodies were incubated overnight at 4 °C. For the secondary fluorescence labeling, the cells were incubated with anti-guinea pig IgG Alexa Fluor 555 (1:200; Abcam, Cambridge, UK) and anti-rabbit IgG Alexa Fluor 488 (1:200; Thermo Fisher Scientific, Waltham, MA, USA). Finally, the cells were stained and mounted with a ProLong gold antifade mountant (Thermo Fisher Scientific, Waltham, MA, USA). The slides were visualized under an EVOS^®^ FL auto cell imaging system (Thermo Fisher Scientific, Waltham, MA, USA).

### 2.8. IPC Clusters in the NF Microwell-Arrayed Membrane Transplantation into Diabetic Nude Mice

The animal experiment was reviewed and approved by the Institutional Animal Care and Use Committee (IACUC No. 2018-12-296, Approval date: 26 December 2018) of the Asan Institute for Life Sciences. The committee abides by the Institute of Laboratory Animal Resources (ILAR) guidelines. Initially, 10^6^ cells were seeded on an NF microwell-arrayed membrane integrated with a custom-designed 12-well insert wall including 165 microwells. In the animal experiment, two NF microwell-arrayed membranes were transplanted to each transplant site. In other words, 330 clusters composed of 2 × 10^6^ cells were transplanted to each animal. First, the cell cultured NF microwell-arrayed membranes were harvested by cutting the membrane out of the insert frame. The membranes with IPC clusters were transplanted to the liver surface, subcutaneous site, and peritoneal wall of 8-week-old male mice. For the liver surface transplantation, the surface of the recipient site was scratched with a dry gauze/cotton swab before transplantation to induce adhesion as described in our previous study [27]. Similar wounds were gently created on the peritoneal wall to enhance membrane attachment. The surface roughness increased, and the transplantation was performed with care to avoid severe bleeding or rupture. The thin NF membranes are easily transplanted and attached to tissue or organ surfaces. Non-transplanted diabetic mice were used as a negative control. In addition, human islets of 2000 IEQ were transplanted into the kidney capsule for use as a positive control. To confirm human insulin secretion after transplantation, human C-peptide from the transplanted IPCs was evaluated using an ultrasensitive human C-peptide ELISA kit (Mercodia, Uppsala, Sweden).

### 2.9. Histological Analysis

After the mice were sacrificed on day 60, the harvested tissues were fixed in 10% formalin solution for 24 h at 4 °C. A paraffin block was prepared with the fixed tissue and cut into 4 μM sections. The samples were deparaffinized, dehydrated, and subjected to staining with hematoxylin and eosin (Sigma Aldrich). Immunohistochemistry was performed using the primary antibodies of rabbit anti-PDX1 and rabbit anti-insulin (dilution 1:200, Abcam, Cambridge, UK). The sections (4 μM thickness) were deparaffinized, dehydrated through a graded alcohol series, blocked with hydrogen peroxide, and dried for 10 min at RT and 20 min in an incubator at 65 °C. An automated slide preparation system (Benchmark XT; Ventana Medical Systems Inc., Tucson, AZ, USA) with the OptiView DAB Detection Kit (Ventana Medical Systems, Tucson, AZ, USA) was used for the immunohistochemistry.

### 2.10. Statistical Analysis

The data are presented as the means ± standard deviation of the mean (SD). A paired 2-tailed t-test was applied to compare the two groups; ANOVA with Tukey multiple comparison tests was used for comparing more than two groups. The sample numbers for each experiment are described in the relevant figure legends. A *p*-value < 0.05 indicates a statistically significant difference.

## 3. Results

### 3.1. Geometric and Permeable Characteristics of the NF Microwell-Arrayed Membrane

**Figure 1:**
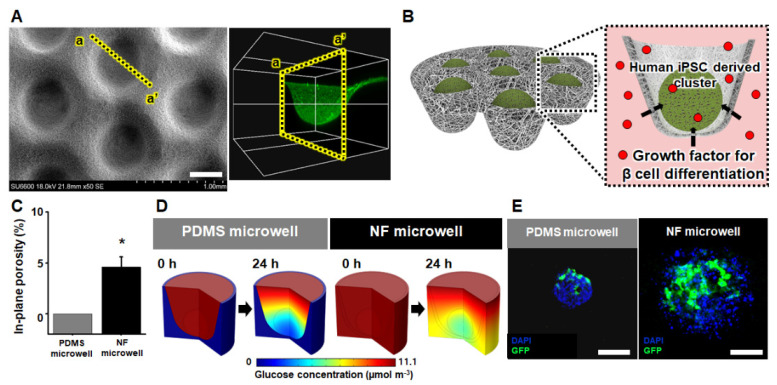
Geometric and permeable characteristics of the NF microwell-arrayed membrane and micro-environment around the human iPSC clusters in the NF microwell. (**A**) SEM image and cross-sectional confocal image of the NF microwell-arrayed membrane, scale bar: 400 µm. (**B**) Scheme of soluble factors permeation through the permeable NF microwell toward the iPSC clusters. (**C**) In-plane porosity of the PDMS impermeable and NF microwell. (**D**) The numerical simulation of the spatial and temporal distribution of the glucose concentration around the iPSC cluster in both the impermeable PDMS and NF microwell. (**E**) GFP expression of cells in the PDMS and NF microwells after transduction of Ad-GFP for 48 h, scale bar: 200 µm. * Represents the statistical difference between PDMS and NF microwell. *p* < 0.05 indicates a significant difference.

As shown in Figure 1A, the matched-mold forming process on the flat NF membrane successfully produced the NF microwell (500 μM in diameter, 250 μM in depth)-arrayed membrane. Figure S2 shows that the NF microwell-arrayed membrane integrated into a custom-designed 12-well insert wall including 165 microwells. The microwell-array structure of the membrane enabled us to collect iPSCs in the microwells and generate iPSC clusters as described in the scheme of Figure 1B. Considering that the most crucial feature of the NF microwell is permeability to soluble factors such as glucose, a growth factor for beta-cell differentiation could be allowed to permeate through the NF membrane toward the iPSC cluster, unlike the existing commercialized PDMS impermeable microwells (Figure 1B). This delivery of soluble factors from the outside of the NF microwell was experimentally demonstrated using diffusional transport through the soluble factors–permeable NF membrane. Figure S3 shows that the red dye solution gradually diffused for 6 h from the basolateral side of the NF microwell-arrayed membrane to the apical side.

The pores, presented in Figure S1, resulted from interconnected nanofibers that allowed the diffusion of soluble factors. The size of the pores was measured from a few micrometers to less than 10 μm as shown in the SEM image of Figure S1, through which cells do not pass, but soluble factors such as nutrients and wastes can permeate. The most crucial reason for the difference between the soluble factors–impermeable PDMS microwell and the permeable NF microwell is the porosity as described in Figure 1C. In detail, the in-plane porosity of the impermeable microwell and the NF microwell was 0 and 0.46, respectively. Figure 1D shows the numerical analysis of the glucose concentration according to the porosity of the PDMS and NF microwell. Because the sides and bottom of the PDMS microwell are impermeable, the nutrients are supplied only from the top. The top side was still rich in nutrients after 24 h, but the bottom side with cell clusters showed a lack of nutrients. However, in the case of the permeable NF microwell, it was found that a certain amount of nutrients was supplied to the bottom after 24 h.

Remarkably, in contrast to the impermeable PDMS microwell, the microenvironment in the NF microwell was found to have a uniform glucose concentration around the iPSC clusters due to diffusional transport through the permeable NF membrane from the basolateral side. To prove these differences in the microenvironment in numerical analysis using another experiment, the human iPSCs utilized in this study were seeded into the PDMS, and NF microwells and clusters were formed after 24 h. The GFP-expressing adenovirus vector was then transduced to the iPSC clusters in both microwells to investigate the GFP expression after 48 h. In the case of the PDMS microwell, the viruses penetrated only the upper surface of the cells and were expressed on one side to the middle because the surrounding wall of the PDMS was impermeable. However, cells expressing GFP were well distributed to the inside of the NF microwell (Figure 1E).

### 3.2. IPC Differentiation from iPSCs Using Microwells

**Figure 2:**
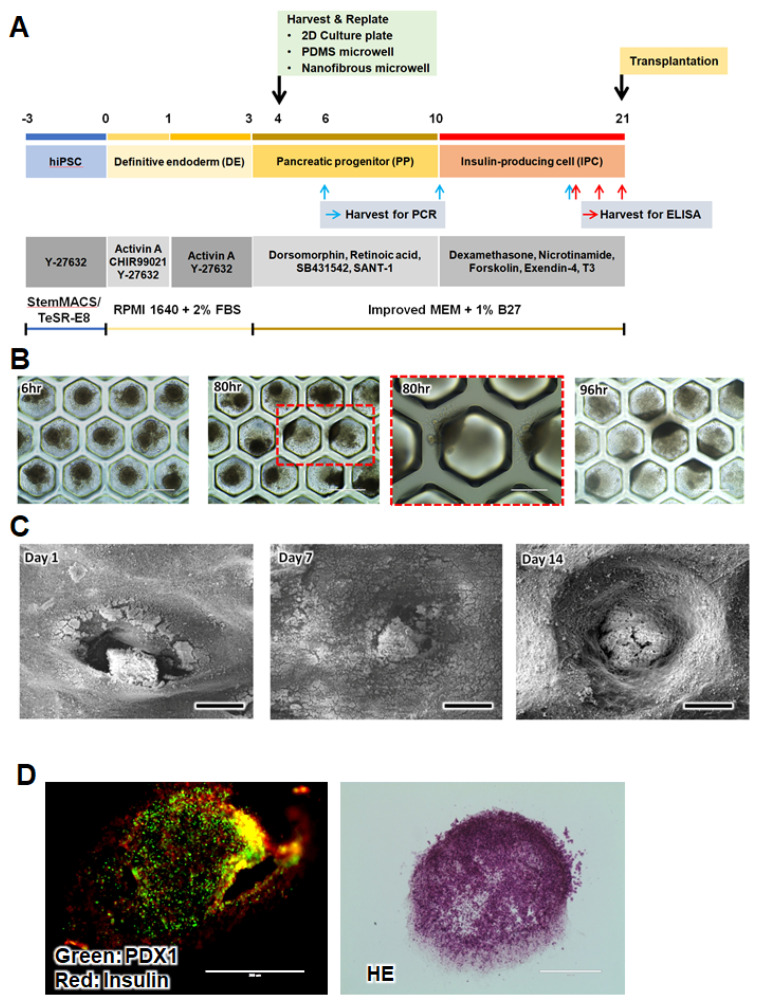
Human iPSC culture and differentiation. (**A**) Overview of the IPC differentiation protocol from iPSCs in 2D culture plates and microwells. The three-stage differentiation protocol including supplements and additives. (**B**) Representative microscopic images of cells in the PDMS microwell at 6, 80, and 96 h after seeding, scale bar: 400 µm (low magnification), 200 µm (high magnification). (**C**) SEM images of cells in the NF microwell at 1, 7, and 14 days after seeding, scale bar: 300 µm. (**D**) Immunohistochemical (PDX1 and insulin) and H&E images of cross-sections of the IPC clusters in the NF microwell on day 21, scale bar: 200 µm.

The differentiation from iPSCs to IPCs was induced in three stages of definitive endoderm (DE), pancreatic progenitor (PP), and insulin-producing cell (IPC) using various growth factors and signaling molecules based on the developmental process of the pancreas (Figure 2A). In the first DE stage of differentiation, many cells died owing to sudden changes in their cell fate and culture conditions. After the DE stage, most of the cells began to proliferate into stable progenitor cells. On day 4, we harvested and replated the cells into new 2D culture plates or microwells. After the induction of differentiation for 3 weeks in vitro, the NF microwell-arrayed membrane containing the IPC cluster was harvested and used for transplantation.

The cluster formation and morphological changes were observed in the NF microwells compared to the PDMS microwells. The IPCs in the impermeable PDMS microwell formed spheroids on day 1, but most of the cells moved toward the sidewall or openings and did not maintain a spherical shape on day 4 (Figure 2B). However, IPCs in the NF microwells remained in clusters within the microwells for up to 2 weeks (Figure 2C,D). After culturing for 21 days, the expression of insulin and PDX1, representative pancreatic transcription factors, could be confirmed by immunostaining of the cells in the NF microwell (Figure 2E).

### 3.3. Comparison of the Differentiation Capacity of the IPCs Culture in 2D Plate, PDMS Microwell, and NF Microwell

**Figure 3:**
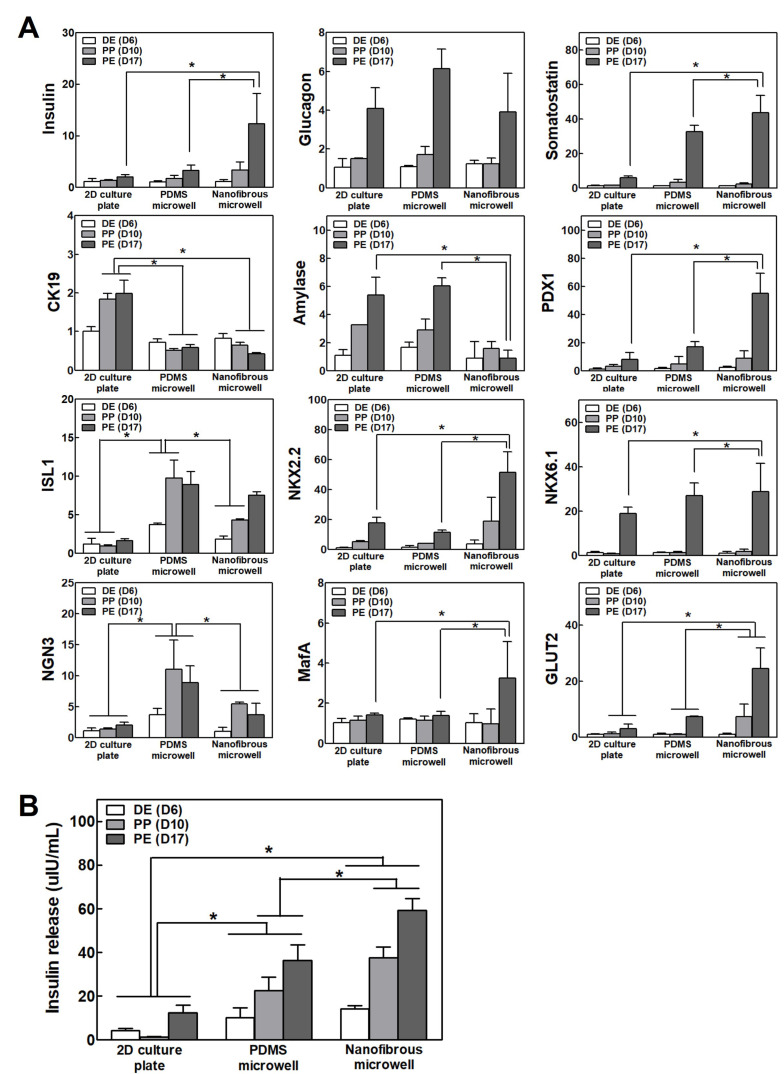
Differentiation efficacy of IPCs in 2D, PDMS microwells, and NF microwells. (**A**) The gene expression of *insulin*, *glucagon*, *somatostatin*, *amylase,* *CK19*, and pancreas-specific transcription factors on days 6, 10, and 17 (n = 4). The results normalized to *GAPDH* gene expression for the same cDNA sample are represented as the relative levels of the mean ± S.D. (**B**) Insulin secretion from IPCs on days 17, 19, and 21 (n = 4). * Represents the statistical difference among the three groups at each time point. *p* < 0.05 indicates a significant difference.

The IPC differentiation capacity under different culture conditions was compared by analyzing the pancreas-related gene expression and insulin secretion. Figure 3A shows the gene expression of the pancreas endocrine markers (*Insulin*, *Glucagon*, and *Somatostatin*), exocrine marker (*Amylase*), duct cell marker (*CK19*), and pancreatic transcription factors on days 6, 10, and 17, which are in the matched differentiation stage. Inducing differentiation from iPSCs into IPCs also leads to differentiation into other related cells present in the pancreatic development process. The pancreas-related gene expression was gradually increased over time with the differentiation in all culture conditions. The IPC clusters in the NF microwells showed the highest expression of insulin and *PDX1*, which is a crucial transcription factor for pancreas development. However, *CK19* and *amylase* expression decreased in the NF microwells, which suggested that cluster formation using the NF microwells can induce differentiated iPSCs into endocrine cells but represses unwanted trans-differentiation into the exocrine and duct. Pancreas-specific transcription factors, including *PDX1*, *ISL1*, *NKX2.2*, and *NGN3*, were increased in the microwell cultures. Remarkably, *MafA* was only expressed in the NF microwell at a late stage, and the highest level of *GLUT2* expression was observed in the NF microwell. Similarly, insulin secretion was significantly increased in 3D microwells relative to the 2D culture condition (Figure 3B).

### 3.4. Transplantation of Permeable NF Microwell-Arrayed Membrane including IPC Clusters

**Figure 4:**
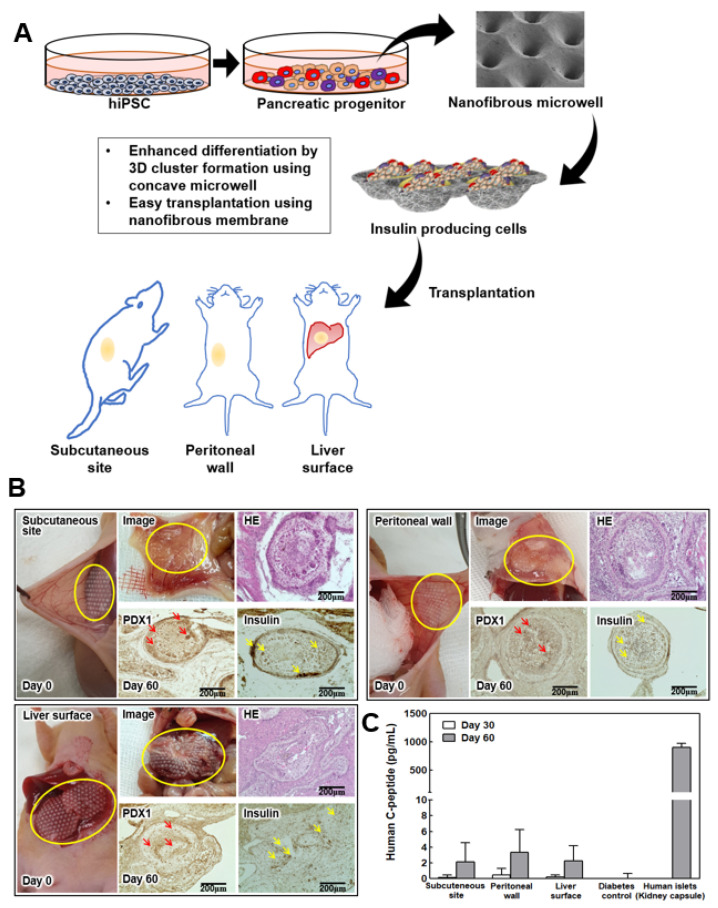
Transplantation of IPC clusters in the NF microwell. (**A**) Schematic description of the experimental procedure. Cells induced to differentiate in the NF microwells. NF membranes including differentiated IPC clusters in the microwell were transplanted for diabetes treatment. (**B**) Optical image on transplantation day; mice transplanted with NF membranes into a subcutaneous site, the liver surface, and peritoneal wall. Transplantation of membranes onto the three sites was performed successfully. Optical image and histology images at 2 months after transplantation. Yellow circle indicates the transplanted NF microwell-arrayed membrane with IPCs. Red arrow indicates PDX1 positive cells. Yellow arrow indicates insulin positive cells, scale bar: 200 µm. (**C**) Human C-peptide levels in blood plasma from mice transplanted with two NF membranes (*n* = 3).

Figure 4A shows the scheme of the experimental procedure of the differentiation and in situ transplantation of the NF microwell-arrayed membranes, including the IPC clusters for diabetes treatment. Thin NF membranes were attached to various organs, including a subcutaneous site, the peritoneal wall, and the liver surface, without any suture or fixation (Figure 4B, photos on day 0). It was found that IPC clusters in the NF microwell engrafted and integrated with the surrounding tissues after 2 months of transplantation (Figure 4B, photos on day 60). Adhesion to the smooth surface of the peritoneum or liver surface could be improved through slight scratches. Through histological evaluation, it was confirmed that the transplanted IPC clusters in the microwells survived well and expressed PDX1 and insulin. Interestingly, the rearrangements of the transplanted cells and blood vessel formation were different according to each transplantation site. To confirm insulin secretion from the transplanted cells, human c-peptide was identified in the blood plasma. As cell differentiation was gradually induced in vivo, higher C-peptide secretion was confirmed at 2 months than at 1 month, but there were no significant differences among the transplantation sites in this study. Human c-peptide was not detected in mice that were not transplanted with cells. For comparison, 2000 IEQ human islets were transplanted into the kidney capsule, and about 900 pg/mL of human c-peptide was detected in this group.

## 4. Discussion

Pancreas islet transplantation is, in theory, an ideal treatment for insulin-dependent diabetes because of the accurate real-time response to the physiological changes of blood glucose, non-invasiveness, and simple application. However, the islets for transplantation can only be obtained from a cadaver pancreas, and it is difficult to obtain and isolate islets from donors for every diabetic patient. Additionally, the transplantation efficiency is not sufficient because of the immune response, and there is also a risk of severe complications from the current transplant method through blood vessel infusion [9]. It is thus necessary to develop a new islet source and transplantation technique as a standard treatment for diabetes patients.

Insulin-producing cells differentiated from stem cells are a potential approach to overcome the clinical application limitations of existing pancreatic islets. Many research groups have reported on technologies and applications related to the differentiation of insulin-producing cells using stem cells [11,28,29]. However, they do not function like a normal pancreas in vivo. The most successful approach to improving their cell function and differentiation capacity is to mimic their natural environment. A unique feature of pancreatic islet cells is that the cells form a spherical shape of 100–300 μM. Owing to the convergence of engineering and biology, cell clusters are easily made in microwell arrays with a uniform size and the desired shape in a manner of mass production [28,29,30,31]. However, conventionally used impermeable microwells are limited by an insufficient supply of nutrients and oxygen to the cells inside this very narrow space, leading to hypoxia or a decrease in their functions. Therefore, in this study, microwells made of porous and permeable NF membranes were developed and applied to solve this problem.

In previous studies, there have been attempts to create and apply a permeable microwell using a porous membrane bottom or a hydrogel [32,33,34]. However, most porous microwells have a rigid frame for drug screening and are impossible to transplant directly. Microwells made only of NF membranes not only have a high permeability as very recently reported [35], but also can be directly transplanted to the target tissue due to the ease of transplanting a flexible, thin, biodegradable, and biocompatible membrane. In this study, we successfully fabricated a permeable NF microwell-arrayed membrane for diabetes treatment by improving upon our previous study [36]. In brief, the microwells are manufactured using a matched-mold forming process to induce cell–cell interactions and maintain the cluster shape of the cultured cells. The previous microwell studies reported that this size and shape are suitable for improving the function of islets.

The diffusional transport of glucose through the NF microwell toward iPSC clusters is important because islets have a physiological function of secreting insulin in response to the glucose concentration. Therefore, we selected glucose as a representative molecule among the various substances in an insulin-producing cell differentiation medium and estimated the glucose concentration around iPSC clusters using a computational simulation method. Glucose was not sufficiently supplied to the cells in the bottom when the cells were cultured in an impermeable microwell for 24 h. In the microwells made of NF membrane, the nutrients were supplied through the pores. The experimental confirmation related to diffusional transport of soluble factors supported the numerical analysis. Additionally, by the transduction of the cells using a direct adenovirus vector, it was shown that the virus could penetrate well into the already formed cluster structure. In the case of PDMS impermeable microwells, clusters were firmly formed, and factors such as nutrients or viruses were supplied only to the top so that only some of them expressed GFP. However, the virus particles penetrated well into the microwell through the pores of the NF membrane.

The IPC differentiation capacity in different culture conditions was compared by analyzing the pancreas-related gene expression and insulin secretion. Inducing differentiation from iPSCs into IPCs also leads to differentiation into other related cells present in the pancreatic development process as well as into insulin-secreting β-cells. The pancreas may be differentiated into other endocrine cells (glucagon secreting α-cells and somatostatin secreting δ-cells), exocrine cells, tubular cells, and so on, and they may also exist as undifferentiated cells. It is necessary to prevent their differentiation into other cells and enhance the differentiation function of the desired insulin-producing cells. Therefore, their differentiation process was evaluated by checking the expression of pancreatic transcription factors other than insulin. The pancreas-related gene expression was gradually increased over time as differentiation proceeded under all culture conditions. The IPC clusters in the NF microwells showed the highest *insulin* and *PDX1* expression, which is a crucial transcription factor for pancreas development. By forming a cell cluster, the cell–cell interaction was maintained, and sufficient differentiation factors and oxygen were supplied through the pores, which improved their differentiation ability. Additionally, *CK19* and *amylase* expression decreased in the NF microwells, suggesting that cluster formation using microwells can induce differentiated iPSCs into endocrine cells and represses unwanted trans-differentiation into the duct or exocrine. Pancreas-specific transcription factors, including *PDX1*, *ISL1*, *NKX2.2*, and *NGN3*, were increased in both microwell cultures compared to 2D cultures. Remarkably, *MafA* was only expressed in the NF microwell at a late stage. In the late stage of pancreatic development, the inhibition of glucagon-secreting alpha cells and the induction of differentiation into insulin-secreting beta cells are significant for enhancing the selectivity and efficiency of differentiation. *MafA* is a critical transcription factor involved in the selective differentiation of beta cells during development and is also involved in the subsequent insulin secretion function [37,38]. Simultaneously, it was confirmed that *GLUT2*, a membrane transporter responsible for recognizing the glucose concentration and secreting insulin from the insulin granules, was expressed at the highest level in the NF membrane cultured cells, thereby improving their insulin secretion ability, which was verified via the insulin secretion experiment.

Currently, most islets are administered directly through the portal vein in clinics because the liver can supply sufficient blood in a near-physiological insulin delivery environment [7,39,40]. However, several concerns remain regarding intraportal islet infusion, including procedure-related complications, bleeding, hepatic hypertension, thrombosis, and immune reactions [16]. Various alternative sites have been proposed, such as the kidney capsule, peritoneal wall, liver surface, omentum, subcutaneous area, and cornea [41]. Although some locations may be advantageous in experimental models, their feasibility and translation into clinical settings are still challenges. The subcutaneous sites have a poor blood supply, the kidney capsule and cornea have limited transplantation space, and special techniques are required to keep cells retained on the liver surface or peritoneal wall [42].

Although intrahepatic injection into the portal vein is widely used for islet transplantation in clinical practice, the injection method could not be used in this study because it was transplanted in the form of a membrane. Because the purpose of this study was to develop a safe and effective local delivery technique of IPCs, its efficacy was evaluated by transplanting the membrane to the liver surface, the peritoneal wall, and the subcutaneous site, all locations that can be used in the clinical trial. Additionally, the kidney capsule is widely used for transplanting cells into animals because it keeps the transplanted cells within the pocket and has abundant blood vessels. However, with this technique, local application of cells in the form of a microwell-arrayed membrane could be possible for any tissue and organs, so we did not use the kidney capsule due to its limited space. Although blood flow is not abundant in our selected sites compared to the kidney, they are considered to be suitable organs for clinical applications. In order to improve the transplantation efficiency, it is thought that additional studies using prevascularization before transplantation should be conducted.

As thin sheets or membranes could attach to various organs without fixation, our developed NF membrane is easily transplanted to these tissues or organ surfaces. On an intact and smooth surface of the peritoneum or liver surface, adhesion could be improved by making slight scratches. In the case of the subcutaneous site, it was transplanted well without any making any effort to improve its adhesion because it was transplanted between the fascia and skin. When the transplant site was checked after animal sacrifice, it was confirmed, through the naked eye and tissue photos, that it was attached and integrated well with the surrounding tissues. Through histological evaluation, it was confirmed that the transplanted cells survived well two months later, differentiated, and secreted insulin. The advantage of the system used here is that the NF microwell-arrayed membrane, including IPC clusters, can be implanted because of PCL’s excellent biocompatibility as approved by the Food and Drug Administration in biomedical applications. In conventional microwells, the cell clusters must be harvested from the microwells and encapsulated in hydrogel for transplantation, or transplantation is only possible in places where pockets could be formed. However, because the microwell developed here can directly implant the NF membrane, there is no need for further cell processing and no limitations on transplantation sites. Furthermore, because nutrients are supplied through the pores as well as the open upper surface owing to the porous NF membrane, in the case of the subcutaneous cells with relatively small blood vessels, small insulin-secreting cells gathered around the permeable membrane, and the formation of a unique, differentiated structure could be confirmed. The liver surface and peritoneal wall had a sufficient blood supply; thus, the transplanted cells were distributed throughout the microwell.

In our previous preliminary experiment, there was a limit to lowering blood glucose when differentiated cells were transplanted. In this study, the efficacy was evaluated by measuring the c-peptide secreted in the blood. In all transplant groups, c-peptide was detected in the mice at 1 month after transplantation, but the level was very low. However, human c-peptide secretion was confirmed in most transplanted animals 2 months after transplantation. The increase in the secretion of insulin or c-peptide in the blood over time compared to a relatively short period after transplantation is thought to be because, although differentiated cells are immature in vitro, the amount of insulin increased as maturation of the differentiated IPCs progressed in vivo. Similar results were also shown in the differentiation of insulin-producing cells from other pluripotent stem cells [43,44]. Compared to the transplantation of human islets into the kidney capsule, the human c-peptide level in the mice with the transplanted differentiated IPCs was still too low to control blood glucose in vivo. The differentiated cells still had a limited efficacy of insulin secretion in vivo relative to human islets. To achieve successful glucose control in clinics, additional studies using a cell source with higher differentiation potential, co-transplantation with other cells, or prevascularization of the implant are necessary. Many studies have been conducted to make insulin-producing cells from stem cells or other cells and use them as a cell therapy for diabetes treatment. However, there are still limitations that have not been reached in making mature differentiated cells with similar efficacy to islet cells in the human pancreas. Therefore, more research is needed to be applied to actual clinical practice. However, given the fact that several leading research groups are currently producing mature differentiated cells, and that stem cells can produce cells with proliferative ability and immunomodulatory functions through genetic manipulation, there is a great hope for the treatment of diabetes mellitus in the future.

## 5. Conclusions

In this study, we successfully developed a differentiation and transplantation technology of insulin-secreting cells for the fundamental treatment of diabetes. For the differentiation of insulin-producing cells, it is essential to form a cell cluster like the pancreatic islet cells. In this study, microwells made of an NF membrane were fabricated. A thin NF membrane was fabricated using a matched-mold forming process with an electrospun membrane. It was observed that nutrients could be sufficiently supplied because it was made of porous nanofibrous networks. Furthermore, it was confirmed that cells’ survival and differentiation functions were improved. The NF membrane was attached to the organ’s surface without any fixing material, and it integrated with the surrounding tissues and secreted insulin. From this, it can be inferred that the NF microwell-arrayed membrane can be a new treatment method as a stem cell differentiation and in situ transplantation platform for curative cell therapy of diabetic patients.

## Figures and Tables

**Table 1:** Primers used for qPCR.

Gene		Sequence (5′ →3′)	Product Size (bp)
*Amylase*	Forward	GGTTCAGGTCTCTCCACCAA	214
	Reverse	TCCTGCACTCACAGCGTTAC	
*CK19*	Forward	AACGGCGAGCTAGAGGTGA	91
	Reverse	GGATGGTCGTGTAGTAGTGGC	
*GAPDH*	Forward	GAAGGTGAAGGTCGGAGT	226
	Reverse	GAAGATGGTGATGGGATTTC	
*Glucagon*	Forward	CCCAAGATTTTGTGCAGTGGTT	221
	Reverse	GCGGCCAAGTTCTTCAACAAT	
*GLUT2*	Forward	AGCTTTGCAGTTGGTGGAAT	300
	Reverse	AATAACAATGCCCGTGACGA	
*Insulin*	Forward	GCAGCCTTTGTGAACCAACAC	67
	Reverse	CCCCGCACACTAGGTAGAGA	
*ISL1*	Forward	ATTTCCCTATGTGTTGGTTGCG	229
	Reverse	CGTTCTTGCTGAAGCCGATG	
*MAFA*	Forward	TTCAGCAAGGAGGAGGTCAT	216
	Reverse	CGCCAGCTTCTCGTATTTCT	
*NEUROD1*	Forward	CCCTGTACACCCCTACTCCT	92
	Reverse	GAGGCTTAACGTGGAAGACA	
*NKX2.2*	Forward	CGGCGAGTGCTTTTCTCCAA	165
	Reverse	GCGCTTCATCTTGTAGCGG	
*NKX6.1*	Forward	CACACGAGACCCACTTTTTC	76
	Reverse	CCGCCAAGTATTTTGTTTCT	
*PDX1*	Forward	GCATCCCAGGTCTGTCTTCT	140
	Reverse	CACTGCCAGAAAGGTTTGAA	
*Somatostatin*	Forward	CTGTCTGAACCCAACCAGAC	90
	Reverse	CAGCTCAAGCCTCATTTCAT	

## Data Availability

The data that support the findings of this study are available on request from the corresponding author.

## Supplementary Materials

The following supporting information can be downloaded at: https://www.mdpi.com/article/10.3390/pharmaceutics14020400/s1, Figure S1: Images investigating the in-plane porosity of the NF microwell. (left) SEM image, (right) Processed image indicating the pores, Scale bar: 4 µm; Figure S2: NF microwell-arrayed membrane integrated in a custom-designed 12-well insert wall; Figure S3. Diffusional transport test through the NF microwell-arrayed membrane using red dye solution.
