# Supplementary material for: Enhanced Differentiation Capacity and Transplantation Efficacy of Insulin-Producing Cell Clusters from Human iPSCs Using Permeable Nanofibrous Microwell-Arrayed Membrane for Diabetes Treatment
Source: Pharmaceutics. 2022 Feb 12;14(2):400. doi: 10.3390/pharmaceutics14020400 (PMC8879814; doi:10.3390/pharmaceutics14020400)
Supplement: Supplementary file 1 [file pharmaceutics-14-00400-s001.zip › pharmaceutics-1524724-supplementary.pdf]

pharmaceutics

Supplementary Materials: Enhanced Differentiation Capacity and Transplantation Efficacy of Insulin-Producing Cell Clusters from Human iPSCs Using Permeable Nanofibrous Microwell-Arrayed Membrane for Diabetes Treatment

In Kyong Shim, Seong Jin Lee, Yu Na Lee, Dohui Kim, Hanse Goh, Jaeseung Youn, Jinah Jang, Dong Sung Kim and Song Cheol Kim

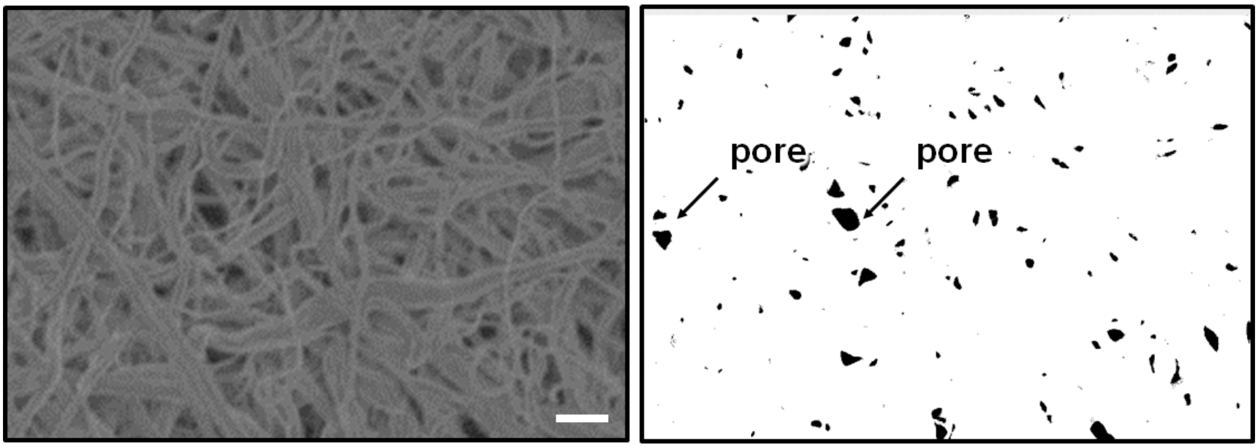

Figure S1. Images in investigating in-plane porosity of the NF microwell. (left) SEM image. (right) Processed image indicating pores,Scale bar:4 μm.

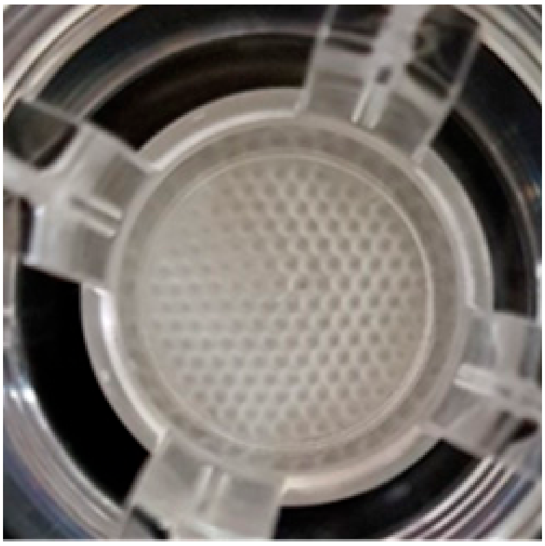

Figure S2. NF microwell-arrayed membrane integrated to a custom-designed 12-well insert wall.

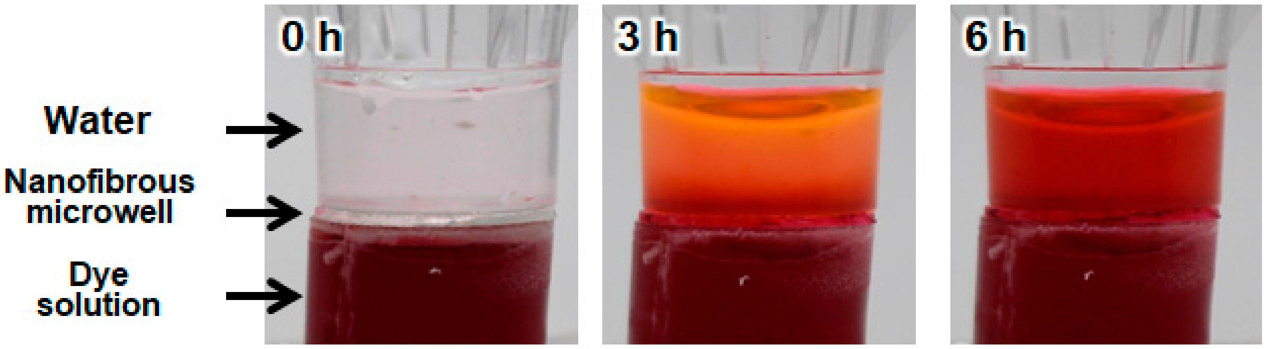

Figure S3. Diffusional transport test through NF microwell-arrayed membrane using red dye solution.
